# Screening for Fatal Traumatic Brain Injuries in Cerebrospinal Fluid Using Blood-Validated CK and CK–MB Immunoassays

**DOI:** 10.3390/biom11071061

**Published:** 2021-07-20

**Authors:** Johann Zwirner, Sven Anders, Simone Bohnert, Ralph Burkhardt, Ugo Da Broi, Niels Hammer, Dirk Pohlers, Rexson Tse, Benjamin Ondruschka

**Affiliations:** 1 Department of Anatomy, University of Otago, Dunedin 9016, New Zealand; 2 Institute of Legal Medicine, University Medical Center Hamburg-Eppendorf, 22529 Hamburg, Germany; s.anders@uke.de; 3 Institute of Legal Medicine, University of Leipzig, 04103 Leipzig, Germany; 4 Institute of Forensic Medicine, University of Wuerzburg, 97078 Wuerzburg, Germany; simone.bohnert@uni-wuerzburg.de; 5 Institute of Clinical Chemistry and Laboratory Medicine, University Hospital Regensburg, 93053 Regensburg, Germany; ralph.burkhardt@ukr.de; 6 Department of Medicine, Forensic Medicine, University of Udine, 33100 Udine, Italy; ugo.dabroi@uniud.it; 7 Institute of Macroscopic and Clinical Anatomy, University of Graz, 8010 Graz, Austria; niels.hammer@medunigraz.at; 8 Department of Trauma, Orthopedic and Plastic Surgery, University Hospital of Leipzig, 04103 Leipzig, Germany; 9 Fraunhofer Institute for Machine Tools and Forming Technology, 09126 Dresden, Germany; 10 Center of Diagnostics, Klinikum Chemnitz, 09116 Chemnitz, Germany; d.pohlers@laborchemnitz.de; 11 Department of Forensic Pathology, LabPLUS, Auckland City Hospital, Auckland 1023, New Zealand; rexsont@adhb.govt.nz

**Keywords:** cerebrospinal fluid, creatine kinase, fatal traumatic brain injury, postmortem biochemistry

## Abstract

A single, specific, sensitive biochemical biomarker that can reliably diagnose a traumatic brain injury (TBI) has not yet been found, but combining different biomarkers would be the most promising approach in clinical and postmortem settings. In addition, identifying new biomarkers and developing laboratory tests can be time-consuming and economically challenging. As such, it would be efficient to use established clinical diagnostic assays for postmortem biochemistry. In this study, postmortem cerebrospinal fluid samples from 45 lethal TBI cases and 47 controls were analyzed using commercially available blood-validated assays for creatine kinase (CK) activity and its heart-type isoenzyme (CK–MB). TBI cases with a survival time of up to two hours showed an increase in both CK and CK–MB with moderate (CK–MB: AUC = 0.788, *p* < 0.001) to high (CK: AUC = 0.811, *p* < 0.001) diagnostic accuracy. This reflected the excessive increase of the brain-type CK isoenzyme (CK–BB) following a TBI. The results provide evidence that CK immunoassays can be used as an adjunct quantitative test aid in diagnosing acute TBI-related fatalities.

## 1. Introduction

Forensic biochemistry is an emerging discipline that provides new insights into death-related systemic pathophysiology [1] and has been found useful in medico-legal investigations such as assisting in determining the cause of death [2,3,4,5] or estimating the time since death [6]. Traumatic brain injury (TBI) is a global health priority due to the growing burden it constitutes in injuries worldwide [7]. Numerous biomarkers have been clinically investigated as markers for diagnostic purposes [8,9] or as outcome predictors [10,11]. A biomarker that can diagnose TBI would be useful, especially in cases where there is an absence of or minimal external head trauma such as in infant head injuries [12] or deceleration injuries from falls [13] or sports-related injuries [14].

Previous postmortem studies showed that central nervous system biomarkers and acute phase proteins were significantly increased in the cerebrospinal fluid (CSF) of TBI fatalities [4,5]. It was recently shown that combining postmortem biomarkers were more accurate for diagnosing TBI compared to a single biomarker [15].

Thus, combining biomarkers may be a reasonable approach as no single, TBI-specific and TBI-sensitive biomarker is available.

Rather than looking for new biomarkers, it would be economically more favorable to investigate established laboratory biomarkers and assays that may not have been associated with TBIs and then use them use in combination with already studied TBI makers [15]. One such biomarker is creatine kinase (CK), and its heart-type isoenzyme CK–MB, which is routinely used in clinical settings to investigate myocardial injury [16]. 

After an impact to the head or intracranial bleeding, the CSF CK level increases as the cytosolic brain-type CK isoenzyme (CK–BB) is released from damaged brain cells (i.e., neurons and astrocytes) [17,18]. Serum and CSF CK–BB elevation is observed a few hours after a TBI [19] and after a subarachnoid hemorrhage respectively [20]. CSF CK–BB is also useful for clinically estimating the degree of brain damage and can be used to assess neurological prognosis following cardiac arrest [21]. In forensic pathology, both the total postmortem CSF CK and CK–BB have shown potential diagnostic utility in only a small number of cases [18,22]. However, postmortem serum CK–BB was unable to differentiate TBIs from control cases. This may be due to cytolysis in the gastrointestinal tract, where CK–BB is also expressed [22]. 

Despite their potential role in TBI, CK–BB assays have limited application because they are not routinely available in clinical settings. A potential biomarker would be CK–MB, another CK isoenzyme, which is routinely available in laboratories. Following a TBI, sustained serum CK–MB elevation caused by sympathetic nervous system stimulation and subsequent myocardial damage was observed in living patients [23]. However, postmortem serum CK and CK–MB are unstable and unsuitable for TBI detection [24]. This limitation was also recognized in studies that attempted to use postmortem serum CK–MB to diagnose cardiac deaths [25,26,27]. Although studies showed postmortem CSF CK–MB unable to diagnose cardiac deaths, no study has investigated its use on TBI-related fatalities [28]. 

The aims of the present study were to explore changes in CSF CK and CK–MB levels in TBI fatalities and assess their diagnostic potential for screening purposes in forensic trauma biochemistry. 

## 2. Materials and Methods

### 2.1. Sample Retrieval

CSF samples were collected during routine forensic autopsies at the Institute of Legal Medicine of the University of Leipzig, Germany. Ethical approval was granted by the Ethics Committee of the University of Leipzig (approval number: 388-15-ek). 

A total of 92 samples were collected and allocated into two groups according to the cause of death. The TBI group consisted of 45 samples with intracranial bleeding following blunt force trauma and macroscopically/microscopically proven cortical contusions. The samples were further divided into three subgroups: 23 acute (survival time < 2 h, median survival time = 8 min, interquartile range (IQR) = 55 min); 12 subacute (survival time 2–72 h; median = 10 h, IQR = 29.5 h); and 10 delayed death (survival time > 72 h; median = 162.5 h, IQR = 165.5 h).

**Figure 1:**
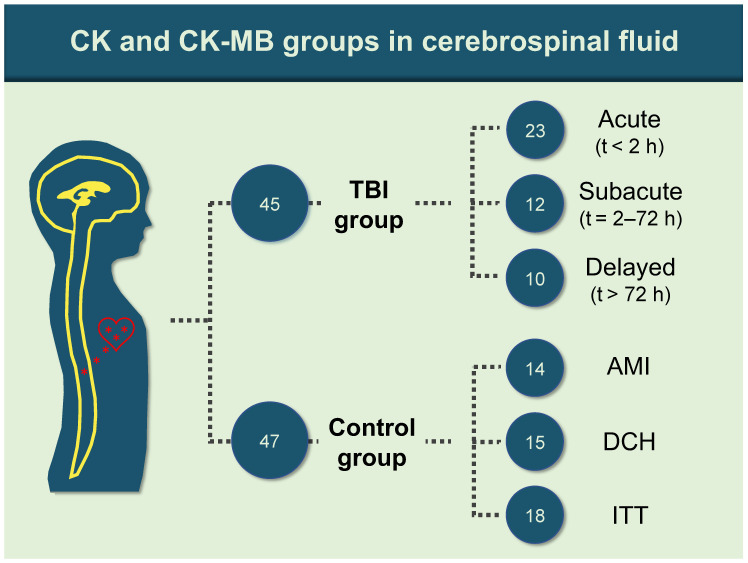
Allocation of postmortem CSF CK and CK–MB samples to TBI and control subgroups (AMI, acute myocardial infarction; DCH, diffuse cerebral hypoxia; h, hours; ITT, isolated torso trauma; TBI, traumatic brain injury; t, survival time).

The control group, 47 samples, was subdivided into acute myocardial infarction (AMI; 14 samples), diffuse cerebral hypoxia (DCH; 15 samples: suffocation, hanging and strangulation), and isolated torso trauma (ITT; 18 samples: blunt and sharp forces to any part of the body excluding the head, predominantly the neck and front side of the trunk—Figure 1).

Survival time for the AMI and DCH group was “non-existent”, as the individuals died immediately following the respective event, whereas the ITT cases showed a median survival time of 60 min (IQR = 65 min). Times were determined on the basis of medical and police records and pathological findings. 

The CSF samples were aspirated from the suboccipital subarachnoid space using a sterile syringe. The CSF samples were centrifuged at 5000 rpm for 5 min at 4 °C and then stored in aliquots at −80 °C until further processed. This allowed reducing the influence of cellular components such as erythrocytes on the biomarker levels. Visible signs of putrefaction and decay, neurodegenerative diseases, neoplasms, and both previous and existent TBIs of the control group samples were excluded from the study. Data regarding age, brain weight, postmortem interval (PMI) and sex are shown in Table 1.

### 2.2. Laboratory Procedures

All the CSF samples were analyzed on a standard automated clinical chemistry analyzer (Cobas 8000, Roche Diagnostics, Mannheim, Germany) using fully automated assays for CK (Roche Diagnostics, Mannheim, Germany, ref. 07190794190, photometry, UV test) and CK–MB (Roche Diagnostics, Mannheim, Germany, ref. 07190808190, immunologic UV test), both certified for in-vitro diagnostics in plasma and serum. The hemolysis index (H index), representing the concentration of free hemoglobin in the sample, was determined by measuring absorbance at 600/570 nm in saline-diluted samples (ref. 05172179190, using the c701 module by Roche Diagnostics, Mannheim, Germany). According to the manufacturers data sheet for the CK and CK–MB immunoassays, an interference of hemolysis can be excluded up to an H index of 20 [29].

### 2.3. Statistical Analysis

Statistical analysis was performed using GraphPad Prism version 8 (GraphPad Software, San Diego, CA, USA) and Microsoft Excel version 16.16 (Microsoft Corporation, Redmond, WA, USA). The Anderson–Darling normality test was used to assess the Gaussian distribution of the data. Receiver operating characteristic (ROC) curve analysis was performed to identify sensitivity and specificity by conservative estimations for differentiation between TBI and controls using the Wilson–Brown method (confidence interval = 95%). Potential threshold values (accentuated for specificity) were determined from the ROC analysis based on maximum positive likelihood ratios with sensitivity values of at least 20%. A Kruskal–Wallis test followed by an uncorrected Dunn’s test was applied to compare subgroups among each other, TBI subgroups to the overall control group and control subgroups to the overall TBI group. The same test was applied to compare confounding parameters between TBI cases and controls and CK/CK–MB levels between TBI cases and controls with H indices < 20. CK/CK–MB levels between the overall TBI and the control group were tested for statistically significant differences using the area under the ROC curve (AUC). Bivariate analyses (Pearson’s r for parametric data and Spearman’s ρ for non-parametric data; two-tailed) were performed among the CK/CK–MB levels, age, brain weight, H index and PMI. A *p* value ≤ 0.05 was considered statistically significant. Medians and IQRs were provided in the text.

## 3. Results

### 3.1. CSF CK and CK–MB Levels Were Significantly Higher in Acute TBIs

**Figure 2:**
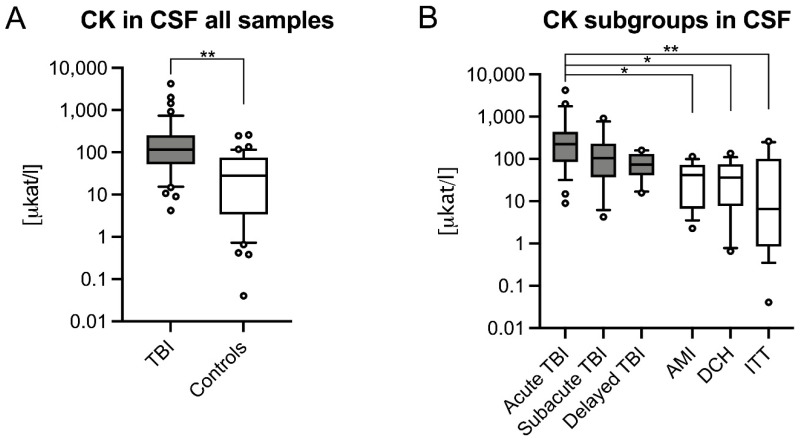
Creatinine kinase (CK) levels in cerebrospinal fluid (CSF) samples of (**A**) all traumatic brain injury (TBI), control cases and (**B**) their respective subgroups are depicted logarithmically. Whiskers indicate the 10th and 90th percentile; circles show outliers. AMI, acute myocardial infarction; DCH, diffuse cerebral hypoxia; ITT, isolated torso trauma; *, *p* ≤ 0.05; **, *p* ≤ 0.001.

CSF CK was significantly higher in TBI (117.5 μkat/l IQR = 200.74 μkat/l) compared to controls (28.28 μkat/l, IQR = 72.32 μkat/l, *p* < 0.001; Figure 2A) and control subgroups (AMI: *p* = 0.027; DCH: *p* = 0.014; ITT: *p* < 0.001). Among TBI subgroups, acute TBI had significantly higher CK (224.1 μkat/l, IQR = 355.84 μkat/l) compared to control (*p* < 0.001), and individual control subgroups (AMI: 41.98 μkat/l, IQR = 66.01 μkat/l, *p* = 0.003; DCH: 36.12 μkat/l, IQR = 68.05 μkat/l, *p* = 0.002; ITT: 6.47 μkat/l, IQR = 99.27 μkat/l, *p* < 0.001; Figure 2B). 

**Figure 3:**
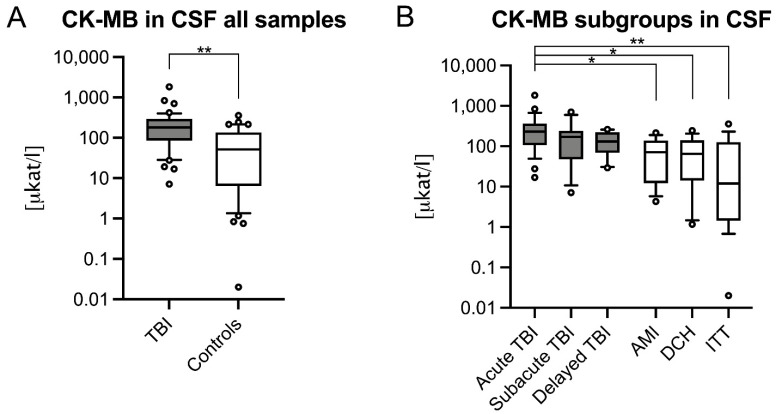
Heart-type creatinine kinase (CK–MB) levels in cerebrospinal fluid (CSF) samples of (**A**) all traumatic brain injury (TBI), control cases and (**B**) their respective subgroups are depicted logarithmically. Whiskers indicate the 10th and 90th percentile; circles show outliers. AMI, acute myocardial infarction; CSF, cerebrospinal fluid; DCH, diffuse cerebral hypoxia; ITT, isolated torso trauma; *, *p* ≤ 0.05; **, *p* ≤ 0.001.

CSF CK–MB was significantly higher in TBI (180.8 μkat/l, IQR = 206.59 μkat/l) compared to controls (51.61 μkat/l, IQR = 128.81 μkat/l, *p* < 0.001; Figure 3A) and in the ITT control subgroup (*p* < 0.001). The CSF CK–MB values often exceeded total CK values which appears implausible. Similar to CSF CK, only the acute TBI CSF CK–MB was significantly higher (229.6 μkat/l, IQR = 258.2 μkat/l) compared to control (*p* < 0.001) and control subgroups (AMI: 71.18 μkat/l, IQR = 126.39 μkat/l, *p* = 0.016; DCH: 64.86 μkat/l, IQR = 126.22 μkat/l, *p* = 0.012; ITT: 11.93 μkat/l, IQR = 125.87 μkat/l, *p* < 0.001; Figure 3B). 

### 3.2. Diagnostic Accuracy of CK/CK–MB Levels in CSF Is Moderate to High

**Figure 4:**
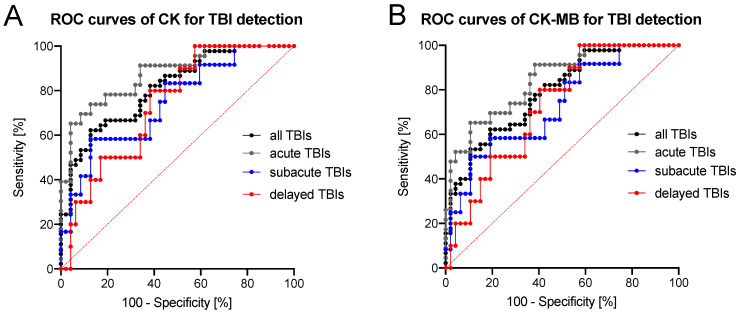
Receiver operating characteristics (ROC) curves are shown for (**A**) creatine kinase (CK) and (**B**) heart-type creatine kinase (CK–MB). Descriptive data to the ROC curves is depicted in Table 2. TBI, traumatic brain injury.

The ROC curve analyses showed that both CSF CK and CK–MB levels were able to differentiate among all TBI fatalities and controls with moderate to high diagnostic accuracy (Figure 4, Table 2). The diagnostic accuracy to distinguish between acute TBIs and controls using CSF levels of CK was high (AUC = 0.876, Figure 4, Table 2). A threshold CSF CK value of 137.2 μkat/l was found differentiating a fatal TBI from other traumatic, cardiovascular or hypoxic causes of death with a sensitivity of 65.2% and a specificity of 95.7% (Table 2). All other TBI subgroups could be distinguished from control cases with moderate diagnostic accuracy based on their CSF CK and CK–MB levels (Figure 4, Table 2).

### 3.3. Quality Control of Samples and Subgroups with Bivariate Analysis Results

The CSF samples were clear on visual inspection in the majority of cases (median H index for CSF = 6, range 0–288), but diminutive traces of blood were unpreventable during sampling. The H index for TBI cases was 41 (IQR = 136) and was significantly higher compared to control cases which had a H index of 2 (IQR = 3, *p* < 0.001). The significant differences between the CSF CK and CK–MB levels of TBI fatalities and control cases persisted even when only cases with a H index of up to 20 were compared, resulting in a comparison of 17 TBI cases and 46 controls (CK: *p* = 0.022; CK–MB: *p* = 0.017). The H index between the TBI subgroups showed no significant statistical difference (*p* ≥ 0.574). The H index was independent of the PMI in both TBI (*p* = 0.372) and control (*p* = 0.545), but moderately correlated with the CSF CK (TBI cases: *p* = 0.010, r = 0.380; controls: *p* = 0.013, r = 0.358) and CSF CK–MB (TBI cases: *p* = 0.005, r = 0.408; controls: *p* = 0.028, r = 0.320). No statistical difference in age, brain weight, sex and PMI were found between TBI and control. However, both the CSF CK (*p* < 0.001, r = 0.507) and CK–MB (*p* < 0.001, r = 0.503) values of the control group revealed a moderate positive correlation with PMI, which was absent in the TBI group (*p* ≥ 0.887). Both CSF CK and CK–MB levels were otherwise independent of age (*p* ≥ 0.502), brain weight (*p* ≥ 0.078) and sex (*p* > 0.069) in both the TBI and control groups.

## 4. Discussion

Postmortem CSF CK–MB had previously been investigated as a biomarker for the cause of death. One study examined 295 fatalities which had 29 TBI cases with only seven cases categorized as acute deaths with survival times of up to three hours [30]. In that study, acute CK–MB CSF levels were shown to be higher when compared to fire- or temperature-related fatalities [30]. In another study of 1923 autopsy cases, TBI was not specifically analyzed [28]. Total CSF CK was investigated in TBI-related fatalities and higher levels were noted compared to non-traumatic hypoxic brain damage, cardiac and miscellaneous causes of death [18,22]. No studies used both CSF CK and CK–MB for analysis and none stratified TBI survival times. 

The results of the study showed that both blood-validated assays for CK and CK–MB can be used in postmortem CSF to discriminate TBI fatalities from control cases with moderate to high diagnostic accuracy. After stratifying the TBI group into different survival times, the overall differences in CSF CK and CK–MB levels between TBI cases and controls came from the acute TBI subgroup (survival time of up to 2 h). Both CSF levels of CK and CK–MB decreased with increasing survival time, resulting in comparable levels between longer survival times of TBI and controls. 

In-vivo CSF samples of healthy individuals are supposed to be free of CK–MB and the muscle-type isoenzyme CK–MM [31]. However, following brain injury, CSF CK–BB and mitochondrial CK increase [18,32]. It has been suggested that CSF CK–MB are influenced by recombination of CK–MM and CK–BB as a consequence of blood contamination [31].

The increased levels of CSF CK following TBI observed in this study could be interpreted as a consequence of either the (traumatic) breakdown of the blood–brain barrier (BBB) by an influx of CK that originated outside of the central nervous system, or an increased release of CK from damaged cells within an intact BBB, or a combination of the two.

### 4.1. Effects of Increased CK Influx into the CSF from Outside the CNS Following Severe TBI

After traumatic injury to the brain, the BBB becomes mechanically disrupted leading to an extravasation of erythrocytes and proteins. This may be reflected macroscopically by scattered petechiae throughout the brain tissue or a yellow-reddish discoloration of the CSF during autopsy [33]. As even large plasma proteins such as albumin can be found in the CSF of TBI patients clinically [34], the mechanical and functional disruption of the BBB [33] can lead to an uncontrolled influx of CK–MM and CK–MB into the CSF. In blood serum, CK in vivo levels in patients with rhabdomyolysis were shown to rise within 12 h and peak within one to three days [35]. Similarly, serum CK–MB levels following myocardial infarctions have a latency time of at least one hour before an elevation can be detected in living patients [36]. Therefore, the survival times of acute TBI fatalities in this study are insufficient to account for the peripheral CK isoenzymes that caused significant elevations in the CSF.

### 4.2. Increase of CK Is Likely Caused by CK–BB Isoenzyme

The acute significant increase of CSF CK following a traumatic impact to the head is probably caused by the release of CK–BB in the cytosol of damaged brain cells, especially neurons and astrocytes [18,37]. It accounts for 80–95% of total CK activity in the brain [18]. An increase in CSF CK–BB is expected in fatal TBIs, and this increase correlated with the amount of brain damage in both human and animal studies [38,39]. Thus, the increase in CK levels of acute TBI fatalities seen in this study was likely the consequence of an increase in brain-specific CK–BB. Given that a specific CK–BB assay is commonly unavailable in routine laboratories, the widely established and economical standard CK enzyme activity assay served as a sufficient substitute in a postmortem setting. Previous studies on postmortem CK–BB used the now discontinued “Impress-BB” kit, which was manufactured by International Immunoassay Laboratories and allowed to directly determine CK–BB activity, obviating interference from CK–MB subunits [18,22]. Nevertheless, the purpose of this study was to determine if standard laboratory assays such as CK and CK–MB were sufficiently accurate to discriminate between TBI fatalities and controls. The results support routine CK and CK–MB assays as potential replacements for the rare and expensive specific CK–BB immunoassays. 

Likewise, acute TBI fatalities could be distinguished from controls by using a commercial CK–MB immuno-inhibition assay to analyze CSF samples. However, CSF CK–MB appears to be influenced by CK–BB to a large degree, and so does not solely reflect the “true” CK–MB levels. Consequently, the CK–MB immunoassay used here served as a qualitative indicator of cerebral damage when applied to the CSF of TBI fatalities. The test principle was originally designed to measure the total catalytic activity of the CK–MB isoenzyme in serum or plasma based on measuring the activity of the CK–B subunit after immuno-inhibition of the CK–M subunit followed by multiplying the result by a factor of two [29]. Regarding the former, it was assumed that the CK–B subunit is generally negligible in blood with higher values, which indicates a malignant disease [40]. Thus, values for the indirectly measured CK–BB can be disproportionally high if it is excessively present in the given fluid, which can be postulated for the fatal TBI cases. This well explains that the measured CK–MB values in this study were often larger compared to the CK values. Additionally, it was impossible to determine the extent to which the CK–MB results reflected the excessive activity of CK–BB or the potential CK–MB activity. Derived from the test principle of the CK–MB immunoassay and assuming the almost exclusive presence of CK–BB in TBI-related CSF samples, the true CK–BB value could potentially be estimated by halving the CK–MB value. However, this remains speculative until it has been validated against the value obtained by a specific CK–BB immunoassay in future research. An electrophoresis evaluation of cadaveric CSF samples for both TBI and non-TBI-related fatalities could further clarify the portion of isoenzymes of the total CK, which could not be performed in the given study due to a lack of material. However, the qualitative activity of the CK isoenzymes could be deduced from this distribution analysis in postmortem samples.

### 4.3. CSF Test Results Are Independent of the Presence of Blood or Contamination

In the study, both CK and CK–MB activity correlated with the H index of the samples, with the latter being significantly larger in TBI fatalities. Both CK and CK–MB levels were also significantly higher in TBI cases when only samples with a maximum H index of 20 were compared as a sub-cohort, for which the used immunoassays were known to be unaffected according to the manufacturer’s data sheet [29]. Increased blood admixtures in CSF samples of TBI fatalities compared to non-TBI-related fatalities were a common finding [5] that can be attributed to the traumatic disruption of blood vessels and the collection of blood in proximity to the CSF, especially in subarachnoid hemorrhages [5]. Hence, an increased H index should be expected when the CSF samples of severe TBIs are processed and not be misinterpreted as contamination due to improper sampling. Any iatrogenic admixture of blood during the sampling should have been avoided or reduced to irrelevance by using the proper centrifugation process before storage. This was underlined by the minute H indices of all control samples.

### 4.4. For TBI Cases, CK Levels in CSF Are Useful for Cause of Death Determinations and Survival Time Estimations but Not for Time since Death Estimations

The results revealed that CK and CK–MB levels in the CSF of TBI fatalities were higher compared to non-TBI-related deaths; therefore, the study hypothesis was confirmed. Interestingly, this was the case even though the CK–MB elevation was most likely attributable to a CK–BB increase in the CSF rather than to an expected response to damaged cardiomyocytes [23] and beyond that to other peripheral interactions after the TBI outside the central nervous system [41]. The diagnostic accuracy for detecting fatal acute TBI in CSF determined with ROC analyses was high for the commercially available standard CK immunoassay and moderate for the commercially available standard CK–MB immunoassay. Consequently, we believe that the results justify adding CK analysis in CSF to forensic biochemistry databases [1] to validate the observations of larger samples, those beyond the 20–88 age range of the study’s TBI group, and to include pediatric samples.

The future inclusion of infant cases, such as inflicted TBI, might help to establish CSF CK as an additional postmortem TBI-related biomarker and support trauma diagnoses by means of forensic biochemistry. Furthermore, CK can be implemented in decision-tree models using several biomarkers to enhance diagnostic accuracy to detect acute, fatal TBI. Our results suggest that a postmortem CSF threshold of 137.2 µkat/l for CK would be adequate to detect acute, fatal TBI irrespective of age, brain weight, sex or PMI. Based on the ROC curve analysis, the measurement of the CSF CK–MB activity using commercial immuno-inhibition assays was inferior compared to the blood-validated total CK assay; therefore, it is not recommended for postmortem TBI-related screening purposes. Furthermore, future studies should investigate how the TBI-related elevation of CK and its isoenzymes relate to the specific TBI subtypes such as epi- or subdural hematomas, intracerebral bleeds or diffuse axonal injuries. 

In summary, the CK level in CSF is a useful supplement for a cause-of-death determination and a TBI survival time estimate in a forensic context, but it is unsuitable for a time-since-death estimate. Moreover, when measuring CK postmortem, a baseline level has to be expected for all CSF samples, which can be taken from CK values in control cases. This is likely caused by either the functional postmortem breakdown of the BBB or the release of CK–BB following the hypoxic–ischemic neuronal death [32] after the oxygen supply to the brain ceases.

CSF CK–BB levels were shown to be 10 to 20 times higher than the last in vivo measurements as soon as 2 h after death [42], which supported our previously stated hypothesis that TBI-related biomarkers in the CSF of fatalities are exceptionally high compared to cases in which the patient survived the trauma [3,5].

### 4.5. Limitations

The study had a limited sample size, and unalterable factors like the environmental temperature during death and storage-related freezing of the CSF samples could have affected degradation and, consequently, biochemical observations. Although considered unlikely for the aforementioned reasons, the observed increase in total CK levels in the CSF could be based on an increase in the isoenzymes CK–MB and CK–MM to an unknown extent, but this could only be excluded by a future specific CK–BB test. Here, an additional CK–BB test would have been advantageous for clarifying whether the TBI-related CK increase was, in fact, caused by an excessive increase of CK–BB, but this was not possible due to limited financial recourses. As CK–MB activity level exceeded values known from clinical investigations, the CK–M subunits might not have been inhibited entirely during the immuno-inhibition and might have contributed to the enzymatic activities for CK as well as for CK–MB. The postmortem influence on a macro-CK formation of CK–MB is unclear, and the measurements might have been affected by this. Furthermore, leakage of peripheral CK isoenzymes due to agony and postmortem changes cannot be excluded. Increased levels of mitochondrial CK were observed in the CSF of patients with hypoxic–ischemic brain damage [32]. Thus, the results in this study might have been influenced by the increased levels of mitochondrial CK in TBI fatalities compared to those of non-TBI fatalities. It should be mentioned that the biomarker levels measured in this study might have been influenced by other injuries related to the traumatic event and might not have been exclusively caused by the fatal TBI. 

## 5. Conclusions

Our data suggested, that elevated CSF levels of CK, determined by a commercial clinical chemistry assay developed for in vitro diagnostics in plasma and serum, may be used to discriminate between fatal acute TBIs and controls. Both CK and CK–MB values seemed to display an excessive increase in CSF CK–BB following trauma-related damage to brain cells. It was suggested that the CK immunoassays can serve as a quantitative postmortem biochemistry test in fatal acute TBI samples. Further studies combining CSF CK and CK–BB to become more established biomarkers for TBI is recommended. 

## Figures and Tables

**Table 1:** Median age, brain weight (m_brain_), postmortem interval (PMI) and sex ratio (interquartile range in parentheses) for the traumatic brain injury (TBI) and control groups and respective subgroups. AMI, acute myocardial infarction; DCH, diffuse cerebral hypoxia; ITT, isolated torso trauma.

	TBI (All)	TBI (<2 h)	TBI (2–72 h)	TBI (>72 h)	Control (All)	AMI	DCH	ITT
**Age (years)**	60 (32.5)	56 (34)	63 (21.5)	52.5 (48.25)	59 (37)	75 (25.5)	53 (30)	51 (49.5)
**m_brain_** **(g)**	1440 (310)	1440 (240)	1423 (259)	1558 (407)	1350 (225)	1340 (193)	1400 (190)	1340 (260)
**PMI** **(hours)**	57 (52.5)	49 (39)	68.5 (75.8)	88.5 (88.3)	52 (39)	57 (19)	53 (33)	21.5 (55.25)
**Males: Females**	39:6	19:4	10:2	10:0	30:17	10:4	10:5	10:8

**Table 2:** Descriptive data to the receiver operator characteristics curves in Figure 4 and results of the specificity-accentuated threshold value analysis. AUC, area under the curve; CK, creatine kinase; CK–MB, heart-type creatine kinase; TBI, traumatic brain injury.

**Group**	**AUC**	**95% Confidence Interval**	**Threshold Value (μkat/l)**	**Sensitivity:** **Specificity** **Ratio (%)**	**Positive** **Likelihood** **Ratio**
**CK in Cerebrospinal Fluid**					
**All TBIs**	0.811	0.726–0.897	137.2	46.7:95.7	10.97
**Acute TBIs**	0.876	0.789–0.963	137.2	65.2:95.7	15.33
**Subacute TBIs**	0.748	0.593–0.904	171.0	33.3:95.7	7.83
**Delayed TBIs**	0.738	0.592–0.884	123.6	30.0:93.6	4.70
**CK–MB in Cerebrospinal Fluid**					
**All TBIs**	0.788	0.698–0.878	246.4	33.3:97.9	15.67
**Acute TBIs**	0.846	0.751–0.940	261.8	47.8:97.9	22.48
**Subacute TBIs**	0.729	0.569–0.888	246.4	25.0:97.9	11.75
**Delayed TBIs**	0.728	0.581–0.875	229.0	20.0:95.7	4.70

## References

[1] Maeda H., Zhu B.-L., Ishikawa T., Quan L., Michiue T. (2009). Significance of postmortem biochemistry in determining the cause of death. Leg. Med..

[2] Zhu B.L., Ishida K., Li Q., Taniguchi M., Oritani S., Li D.R., Fujita M.Q., Maeda H. (2002). Postmortem serum uric acid and creatinine levels in relation to the causes of death. J. Forensic Sci..

[3] Ondruschka B., Pohlers D., Sommer G., Schober K., Teupser D., Franke H., Dressler J. (2013). S100B and NSE as Useful Postmortem Biochemical Markers of Traumatic Brain Injury in Autopsy Cases. J. Neurotrauma.

[4] Ondruschka B., Schuch S., Pohlers D., Franke H., Dreßler J. (2018). Acute phase response after fatal traumatic brain injury. Int. J. Leg. Med..

[5] Ondruschka B., Sieber M., Kirsten H., Franke H., Dreßler J., Dressler J. (2018). Measurement of Cerebral Biomarkers Proving Traumatic Brain Injuries in Post-Mortem Body Fluids. J. Neurotrauma.

[6] Peyron P.-A., Lehmann S., Delaby C., Baccino E., Hirtz C. (2019). Biochemical markers of time since death in cerebrospinal fluid: A first step towards “Forensomics”. Crit. Rev. Clin. Lab. Sci..

[7] James S.L., Theadom A., Ellenbogen R.G., Bannick M.S., Montjoy-Venning W., Lucchesi L.R., Abbasi N., Abdulkader R., Abraha H.N., Adsuar J.C. (2019). Global, regional, and national burden of traumatic brain injury and spinal cord injury, 1990–2016: A systematic analysis for the Global Burden of Disease Study 2016. Lancet Neurol..

[8] Woodcock T., Morganti-Kossmann M.C. (2013). The Role of Markers of Inflammation in Traumatic Brain Injury. Front. Neurol..

[9] Zetterberg H., Blennow K. (2016). Fluid biomarkers for mild traumatic brain injury and related conditions. Nat. Rev. Neurol..

[10] Ferreira L.C.B., Regner A., Miotto K.D.L., De Moura S., Ikuta N., Vargas-Seymour A., Chies J., Simon D. (2014). Increased levels of interleukin-6, -8 and -10 are associated with fatal outcome following severe traumatic brain injury. Brain Inj..

[11] Lustenberger T., Kern M., Relja B., Wutzler S., Störmann P., Marzi I. (2016). The effect of brain injury on the inflammatory response following severe trauma. Immunobiology.

[12] Case M.E. (2008). Inflicted Traumatic Brain Injury in Infants and Young Children. Brain Pathol..

[13] Hellewell S.C., Ziebell J., Lifshitz J., Morganti-Kossmann C. (2016). Impact Acceleration Model of Diffuse Traumatic Brain Injury. Methods Mol. Biol..

[14] Barth J.T., Freeman J.R., Broshek N.K., Varney R.N. (2001). Acceleration-Deceleration Sport-Related Concussion: The Gravity of It All. J. Athl. Train..

[15] Zwirner J., Bohnert S., Franke H., Garland J., Hammer N., Möbius D., Tse R., Ondruschka B. IL-6 and GFAP as a compelling biomarker combination to detect lethal acute traumatic brain injuries in cerebrospinal fluid.

[16] Aydin S., Ugur K., Aydin S., Sahin I., Yardim M. (2019). Biomarkers in acute myocardial infarction: Current perspectives. Vasc. Health Risk Manag..

[17] Rabow L., DeSalles A.A.F., Becker D.P., Yang M., Kontos H.A., Ward J.D., Moulton R.J., Clifton G., Gruemer H.D., Muizelaar J.P. (1986). CSF brain creatine kinase levels and lactic acidosis in severe head injury. J. Neurosurg..

[18] Vazquez M.D., Sánchez-Rodríguez F., Osuna E., Diaz J., Cox D.E., Pérez-Cárceles M.D., Martinez P., Luna A., Pounder D.J. (1995). Creatine Kinase BB and Neuron-Specific Enolase in Cerebrospinal Fluid in the Diagnosis of Brain Insult. Am. J. Forensic Med. Pathol..

[19] Kaste M., Hernesniemi J., Somer H., Hillbom M., Konttinen A. (1981). Creatine kinase isoenzymes in acute brain injury. J. Neurosurg..

[20] Coplin W.M., Longstreth W.T., Lam A.M., Chandler W.L., Mayberg T.S., Fine J.S., Winn H.R. (1999). Cerebrospinal fluid creatine kinase-BB isoenzyme activity and outcome after subarachnoid hemorrhage. Arch. Neurol..

[21] Tirschwell D.L., Longstreth W.T., Rauch-Matthews M.E., Chandler W.L., Rothstein T., Wray L., Eng L.J., Fine J., Copass M.K. (1997). Cerebrospinal fluid creatine kinase BB isoenzyme activity and neurologic prognosis after cardiac arrest. Neurology.

[22] Osuna E., Pérez-Cárceles M., Luna A., Pounder D. (1992). Efficacy of cerebro-spinal fluid biochemistry in the diagnosis of brain insult. Forensic Sci. Int..

[23] Hackenberry L., Miner M., Rea G.L., Woo J., Graham S.H., Hackenberry L., Miner M., Rea G.L., Woo J., Graham S.H. (1982). Biochemical evidence of myocardial injury after severe head trauma. Crit. Care Med..

[24] Woydt L., Bernhard M., Kirsten H., Burkhardt R., Hammer N., Gries A., Dreßler J., Ondruschka B. (2018). Intra-individual alterations of serum markers routinely used in forensic pathology depending on increasing post-mortem interval. Sci. Rep..

[25] Palmiere C., Mangin P. (2012). Postmortem chemistry update part II. Int. J. Leg. Med..

[26] Zhu B.-L., Ishikawa T., Michiue T., Li D.-R., Zhao N., Bessho Y., Kamikodai Y., Tsuda K., Okazaki S., Maeda H. (2007). Postmortem cardiac troponin I and creatine kinase MB levels in the blood and pericardial fluid as markers of myocardial damage in medicolegal autopsy. Leg. Med..

[27] Bañón R., Hernández-Romero D., Navarro E., Pérez-Cárceles M.D., Noguera-Velasco J.A., Osuna E. (2019). Combined determination of B-type natriuretic peptide and high-sensitivity troponin I in the postmortem diagnosis of cardiac disease. Forensic Sci. Med. Pathol..

[28] Chen J.-H., Inamori-Kawamoto O., Michiue T., Ikeda S., Ishikawa T., Maeda H. (2015). Cardiac biomarkers in blood, and pericardial and cerebrospinal fluids of forensic autopsy cases: A reassessment with special regard to postmortem interval. Leg. Med..

[29] (2009). Roche Diagnostics.

[30] Wang Q., Michiue T., Ishikawa T., Zhu B.-L., Maeda H. (2011). Combined analyses of creatine kinase MB, cardiac troponin I and myoglobin in pericardial and cerebrospinal fluids to investigate myocardial and skeletal muscle injury in medicolegal autopsy cases. Leg. Med..

[31] Chandler W.L., Clayson K.J., Longstreth W.T., Fine J.S. (1984). Creatine kinase isoenzymes in human cerebrospinal fluid and brain. Clin. Chem..

[32] Chandler W.L., Clayson K.J., Longstreth W.T., Fine J.S. (1986). Mitochondrial and MB Isoenzymes of Creatine Kinase in Cerebrospinal Fluid from Patients with Hypoxic–Ischemic Brain Damage. Am. J. Clin. Pathol..

[33] Chodobski A., Zink B.J., Szmydynger-Chodobska J. (2011). Blood–Brain Barrier Pathophysiology in Traumatic Brain Injury. Transl. Stroke Res..

[34] Lindblad C., Nelson D.W., Zeiler F., Ercole A., Ghatan P.H., Von Horn H., Risling M., Svensson M., Agoston D.V., Bellander B.-M. (2020). Influence of Blood–Brain Barrier Integrity on Brain Protein Biomarker Clearance in Severe Traumatic Brain Injury: A Longitudinal Prospective Study. J. Neurotrauma.

[35] Subramanian A., Sukheeja D., Trikha V., Pandey A.K., Albert V., Pandey R.M. (2013). Evaluation of Serum Creatine Kinase and Urinary Myoglobin as Markers in Detecting Development of Acute Renal Failure in Severely Injured Trauma Patients. ISRN Emerg. Med..

[36] Cabaniss C.D., Walker H.K., Hall W.D., Hurst J.W. (1990). Creatine Kinase. Clinical Methods: The History, Physical, and Laboratory Examinations.

[37] Thompson R., Kynoch P.A., Sarjant J. (1980). Immunohistochemical localization of creatine kinase-BB isoenzyme to astrocytes in human brain. Brain Res..

[38] Anagnostopoulos D.I., Dontas I.A., Kotsarelis D.V., Julien G., Karayannacos P.E., Diakolios C.E., Skalkeas G.D. (1988). Creatine Kinase (CK–BB) Determination in Cerebrospinal Fluid After Acute Experimental Head Injury. Br. J. Neurosurg..

[39] Hans P., Born J.D., Chapelle J.-P., Milbouw G. (1983). Creatine kinase isoenzymes in severe head injury. J. Neurosurg..

[40] Zarghami N., Yu H., Diamandis E., Sutherland D.J. (1995). Quantification of creatine kinase BB isoenzyme in tumor cytosols and serum with an ultrasensitive time-resolved immunofluorometric technique. Clin. Biochem..

[41] McDonald S., Sharkey J.M., Sun M., Kaukas L.M., Shultz S.R., Turner R.J., Leonard A.V., Brady R.D., Corrigan F. (2020). Beyond the Brain: Peripheral Interactions after Traumatic Brain Injury. J. Neurotrauma.

[42] Karkela J.T. (1993). Critical evaluation of postmortem changes in human autopsy cisternal fluid. Enzymes, electrolytes, acid-base balance, glucose and glycolysis, free amino acids and ammonia: Correlation to total brain ischemia. J. Forensic Sci..

